# Estimating the Asymptomatic Ratio of Norovirus Infection During Foodborne Outbreaks With Laboratory Testing in Japan

**DOI:** 10.2188/jea.JE20170040

**Published:** 2018-09-05

**Authors:** Fuminari Miura, Ryota Matsuyama, Hiroshi Nishiura

**Affiliations:** 1 Graduate School of Medicine, Hokkaido University, Hokkaido, Japan; 2 Department of Urban Engineering, Graduate School of Engineering, The University of Tokyo, Tokyo, Japan; 3 CREST, Japan Science and Technology Agency, Saitama, Japan

**Keywords:** asymptomatic ratio, subclinical infection, *Caliciviridae*, Norwalk virus, statistical estimation, mathematical model

## Abstract

**Background:**

Foodborne norovirus outbreak data in Japan from 2005–2006, involving virological surveillance of all symptomatic and asymptomatic individuals, were reanalyzed to estimate the asymptomatic ratio of norovirus infection along with the risk of infection and the probability of virus shedding.

**Methods:**

Employing a statistical model that is considered to capture the data-generating process of the outbreak and virus surveillance, maximum likelihood estimation of the asymptomatic ratio was implemented.

**Results:**

Assuming that all norovirus outbreaks (*n* = 55) were the result of random sampling from an identical distribution and ignoring genogroup and genotype specificities, the asymptomatic ratio was estimated at 32.1% (95% confidence interval [CI], 27.7–36.7). Although not significant, separate estimation of the asymptomatic ratio of the GII.4 genotype appeared to be greater than other genotypes and was estimated at 40.7% (95% CI, 32.8–49.0).

**Conclusion:**

The present study offered the first explicit empirical estimates of the asymptomatic ratio of norovirus infection in natural infection settings. The estimate of about 30% was consistent with those derived from volunteer challenge studies. Practical difficulty in controlling GII.4 outbreaks was supported by the data, considering that a large estimate of the asymptomatic ratio was obtained for the GII.4 genotype.

## INTRODUCTION

Noroviruses are an evolutionarily diverse group of single-stranded positive-sense RNA viruses without the envelope, belonging to the *Caliciviridae* family, and are responsible for a substantial part of acute viral gastroenteritis in humans.^1^^,^^2^ Norovirus outbreaks have happened both in developing and industrialized nations.^3^ Once infected, symptoms are characterized by non-bloody diarrhea, vomiting, nausea, and abdominal cramps, and there has been no specific treatment to accelerate the cure of patients infected with the virus.^4^ Vaccines have yet to be fully developed and brought into practice,^5^ and the presence of various genotypes prevents infected human from acquiring sufficient specific immunity.^6^

Published studies have reported that around 30% of norovirus infection remains asymptomatic,^7^ while such asymptomatically infected individuals are known to excrete substantial volume of viruses.^8^^,^^9^ Vomit from infectious individuals contains over 10^7^ copies/gram of noroviruses,^8^^,^^9^ and infectious vomitus is known to be sometimes aerosolized,^10^^,^^11^ making it difficult to prevent secondary transmissions. The virus spreads through fecal-oral routes, such as via contaminated water, food, and people’s contaminated hands.^12^^,^^13^ Published studies indicate that infections associated with environmental contaminants are mainly caused by contaminated water or foods (especially oysters),^11^ accounting for seasonal outbreaks that are frequently observed in winter.^14^ Given these features, workers who are directly engaged in cooking and handling of foods, such as cooks, food servers, and food factory workers, are considered as one of the key subjects for prevention of secondary transmissions.^15^^–^^17^

One of the notable virological features of noroviruses is genotype variation, which is divided into five genogroups and many genotypes in each genogroup.^18^ As of 2017, the most prevalent genotype in humans is GII.4,^19^^,^^20^ which accounts for as many as 80% of all reported norovirus infections with virus isolation.^21^ Some pieces of evidence indicate that this genotype evolves rapidly, escaping from selection pressure.^22^^,^^23^ In Japan, the majority of outbreaks are caused by genogroup II, and a large proportion is caused by GII.4.^24^ GII.4-associated epidemics in Japan were first observed in 2006, and the genotype has been continuously observed every year since then.^25^

To decipher the most effective preventive measures against this virus, it is vital to quantitatively clarify the natural history characteristics, including asymptomatic ratio, the risk of infection per exposure, and the probability of virus shedding. Nevertheless, explicit estimates are very scarce, except for rigorous challenge studies that helped quantify the natural history^5^^,^^26^; moreover, it is unclear if the natural history of experimentally infected individuals are similar to those based on natural infection. A small number of mathematical modelling studies estimated a part of the abovementioned values,^27^^–^^30^ but explicit model-based estimates of the asymptomatic ratio have yet to be offered. The present study aims to estimate the asymptomatic ratio of norovirus infection, reanalyzing foodborne outbreak data with laboratory testing in Japan, along with other parameters, including virus shedding frequency and the risk of infection. In addition to estimating the asymptomatic ratio for all noroviruses, the present study also compares the estimate across different genogroups and genotypes.

## METHODS

### Outbreak series data

**Figure 1.**
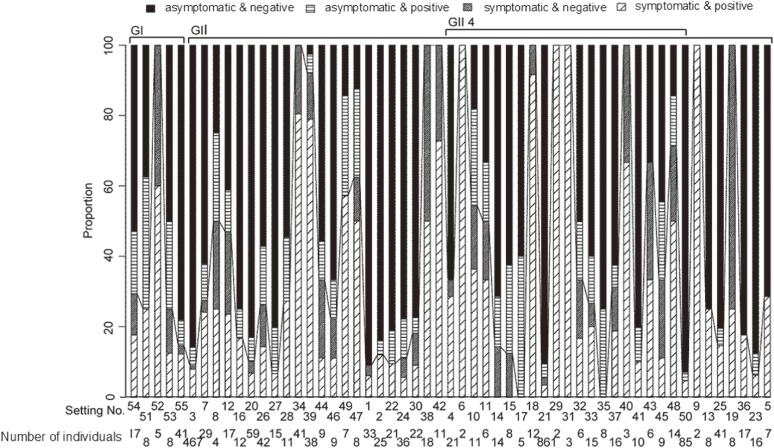
Symptomatic and asymptomatic individuals during foodborne norovirus-outbreaks in Japan from 2005 through 2006. Extracted from Ozawa et al (2007).^31^ Setting No. corresponds to identity numbers given by original research. No. of individuals stands for the total number of individuals diagnosed by stool specimen test at each outbreak setting. Left five bars show the genogroup I (GI)-associated outbreaks. Other 50 bars show the genogroup II (GII)-associated outbreaks, and middle of them emphasize outbreaks caused by the genogroup II type 4 (GII4) norovirus, which is the most dominant among genotypes extracted from acute gastroenteritis patients.

We reanalyzed a published dataset that tested both symptomatic and asymptomatic individuals during norovirus outbreaks at food catering settings in Japan from November 10, 2005 to December 9, 2006 (*n* = 55 outbreaks).^31^ In that study, a total of 2,229 involved individuals, including food-handlers, other workers, and potentially exposed customers, were examined for the presence of symptoms (ie defined as either non-bloody diarrhea or vomiting) and also virus shedding from their feces, conducting a survey shortly after each outbreak (within 1 week). Asymptomatic individuals were defined as persons without diarrhea and vomiting, and the asymptomatic ratio was defined as the proportion of asymptomatically infected individuals among the total of infected individuals. Through stool specimen tests using highly sensitive real-time reverse transcription polymerase chain reaction (RT-PCR), which has the highest detection limit among all existing genome testing methods^32^ the virus and its genogroup/genotype were identified. Due to its highly sensitive nature, the real-time RT-PCR was shown to detect positive norovirus specimens if >10 copies were included in a well.^32^ Since asymptomatic healthy individuals as well as symptomatic cases were tested, the results in each outbreak are classified into four categories, ie, (i) symptomatic and virus positive, (ii) symptomatic but virus negative, (iii) asymptomatic and virus positive, and (iv) asymptomatic and virus negative. In addition to the number of people in each category for each outbreak, genotyping results were obtained. Figure 1 summarizes the survey results, and the coded data are given in eTable 1. It should be noted that the number of asymptomatic and virus-negative individuals includes both uninfected individuals and infected cases without virus shedding. The abovementioned study^31^ has been very rigorous, in that all individuals involved in each food-borne outbreak were fully surveyed, which has been extremely rare and not routinely attainable. In addition, such a study must employ the highly sensitive real-time RT-PCR, a condition that was also satisfied by the 2005–2006 study.^31^ Moreover, all events were observed in the same year in Japan within food handling facilities of comparable sizes.

### Statistical modelling

**Figure 2.**
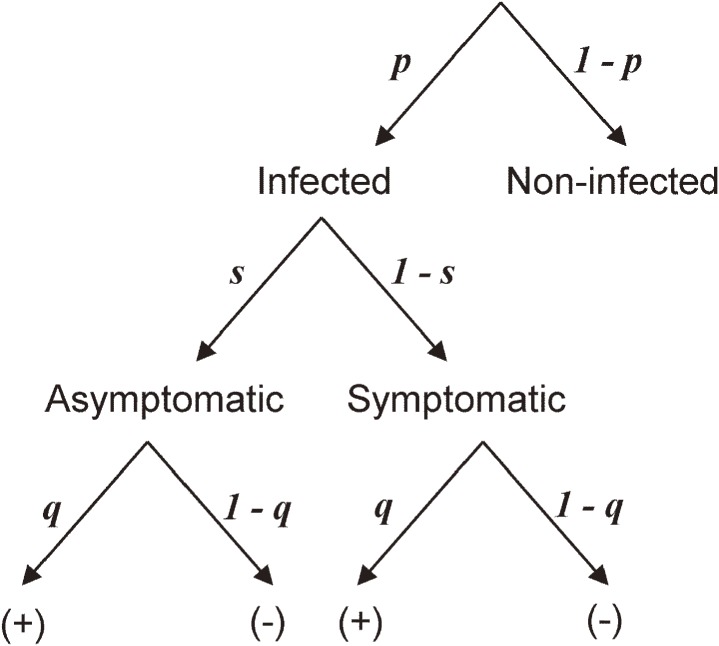
Data generation process of infection, symptomatic illness and virus testing results during norovirus outbreaks in Japan. (+) and (−) represent virus testing result of stool specimens being positive and negative, respectively. *p*, *s* and *q* represent the probabilities of infection, asymptomatic infection given infection, and test positive outcome, respectively.

Here, we describe a statistical model with which the asymptomatic ratio and other parameters were jointly estimated using the abovementioned foodborne outbreak data. First, the data generating process is schematically illustrated in Figure 2. Three pieces of information are considered: the infection process, the illness onset, and virus shedding results. We assume that all situations and population were similar across outbreaks and shared identical parameters. Let *p* be the risk (or the probability) of norovirus infection given an exposure at food handling center, and similarly, let *s* be the asymptomatic ratio of norovirus infection, representing the probability that infected individuals escape from symptomatic illness.^33^ Moreover, let *q* be the probability of virus detection from an infected individual. While these parameters are allocated, all these processes are not directly observed in the original survey. The epidemiological information we have an access includes (i) the total number of people involved (including healthy individuals) in each outbreak, (ii) the number of symptomatic cases, and (iii) the numbers of symptomatic virus shedding cases and asymptomatic virus shedding cases. From these pieces of information, we estimate *p*, *q* and *s* jointly.

Let *i* be an identity of an outbreak (ie, *i* ranges from 1 to 55). All three parameters were dealt with as deterministic parameters; we did not consider demographic stochasticity in the data generating process. Since observed processes are the single and independent success/failure process, the resulting process is a Bernoulli trial, and thus, we assume that each observed event was a result of binomial sampling (because every observed step was binary and mutually independent). The probability that *n*_i_ cases experience symptomatic illness among a total of *n*_i_ symptomatic and *m*_i_ asymptomatic individuals is described by

$$p_{1}(n_{i},m_{i};p,s)=\left(\begin{array}{c} n_{i}+m_{i}\\ n_{i} \end{array}\right){\left(p(1-s)\right)}^{n_{i}}{\left(1-p(1-s)\right)}^{m_{i}}.$$ (1)

Similarly, the probability that *y*_i_ cases are asymptomatic but positive among a total of *m*_i_ asymptomatic individuals (that include uninfected individuals) is

$$p_{2}(y_{i},m_{i};p,q,s)=\left(\begin{array}{c} m_{i}\\ y_{i} \end{array}\right){\left(\frac{psq}{1-p+ps}\right)}^{y_{i}}{\left(1-\left(\frac{psq}{1-p+ps}\right)\right)}^{m_{i}-y_{i}}.$$ (2)

The probability that *x*_i_ cases shed the virus among a total of *n*_i_ symptomatic cases is

$$p_{3}(x_{i},n_{i};q)=\left(\begin{array}{c} n_{i}\\ x_{i} \end{array}\right)q^{x_{i}}{\left(1-q\right)}^{\left(n_{i}-x_{i}\right)}.$$ (3)

It should be noted that *q* was assumed as independent of symptoms because the RT-PCR testing employed was highly sensitive and the cut-off value must have allowed *q* to be comparable between symptomatic and asymptomatic infected individuals.^8^^,^^26^ Sensitivity analysis was conducted to address potential difference of *q* between symptomatic and asymptomatic infection (see below). It should also be noted that the parameter *p* should have ideally reflected the demographic stochasticity of the data generating process, but *p* is a mixture of food-borne and human-to-human transmissions and we do not have a sufficient dataset to characterize the distribution. Thus, the abovementioned process in principle adopted a deterministic modeling approach and measured the uncertainty of *p* based on sampling error alone.

### Maximum likelihood estimation

A maximum likelihood method was employed to estimate parameter values, *p*, *q* and *s*. These parameters were estimated assuming several different interpretation scenarios of empirical data. First, a single set of *p*, *q*, and *s* was estimated, assuming that each outbreak was a random sampling result from the abovementioned mechanisms (1)–(3). Second, parameters were assumed to be different between genogroup 1 and 2, so there were six unknown parameters. Third, parameters were assumed to be different by genotype. When estimating parameters by genotype, it should be noted that the available number of outbreaks differed by genotype (eg, seven outbreaks for GII.3), and we excluded outbreaks in which multiple genotypes were identified. To identify any improved fit despite increased number of parameters, Akaike Information Criterion (AIC) was computed for each scenario.

Using (1)–(3), we derive the total likelihood *L* for each single interpretation scenario, computed as

$$L(p,q,s;data)=L_{1}L_{2}L_{3},$$ (4)

where

$$\{\begin{array}{l} L_{1}(p,s;n,m)=\prod_{i}\left(\begin{array}{c} n_{i}+m_{i}\\ n_{i} \end{array}\right){\left(p(1-s)\right)}^{n_{i}}{\left(1-p(1-s)\right)}^{m_{i}},\\ L_{2}(p,q,s;m,y)\\ \;=\prod_{i}\left(\begin{array}{c} m_{i}\\ y_{i} \end{array}\right){\left(\frac{psq}{1-p+ps}\right)}^{y_{i}}{\left(1-\left(\frac{psq}{1-p+ps}\right)\right)}^{m_{i}-y_{i}},\\ L_{3}(q;n,x)=\prod_{i}\left(\begin{array}{c} n_{i}\\ x_{i} \end{array}\right)q^{x_{i}}{\left(1-q\right)}^{\left(n_{i}-x_{i}\right)}. \end{array}$$ (5)

Maximum likelihood estimates of *p*, *q*, and *s* were obtained by minimizing the negative logarithm of (4), and the 95% confidence intervals (CI) were computed using the profile likelihood.

### Sensitivity analysis

To analyze the sensitivity of parameter estimates to differential frequency of virus shedding between symptomatic and asymptomatic infections, we used the following α, ie,

$$\alpha =\frac{q_{0}}{q_{1}},$$ (6)

where *q*_0_ is the probability of virus shedding from an asymptomatic case and *q*_1_ is the probability from a symptomatic case. We set the baseline value of α as 1.00, which indicates that asymptomatic and symptomatic cases have the same probability of virus shedding (due to a highly sensitive PCR testing method^8^^,^^26^), which is supported by empirical observation.^31^^,^^32^ However, as part of sensitivity analysis, the value of α was changed from 0.50 to 1.00, anticipating that virus shedding frequency from asymptomatic cases might potentially be smaller than that from symptomatic cases.

## RESULTS

When all 55 outbreaks were used and regarded as random sampling results from a single set of parameters, model parameters *p*, *s*, and *q* were estimated as 25.4% (95% CI, 23.4–27.4%), 32.1% (95% CI, 27.7–36.7%) and 73.2% (95% CI, 68.6–77.4%), respectively. eFigure 1 compares the maximum likelihood estimates of this scenario against others. Asymptomatic ratio *s* estimated by genogroups and by genotypes did not reveal any significant difference from combined result, with estimated values ranging from 22.2–66.7% (Table 1). The risk of infection of GI might be slightly greater than that of GII, although the difference was not statistically significant. Among the GII genotypes, GII.4 yielded the highest estimate of asymptomatic ratio (40.7%; 95% CI, 32.8–49.0%) and the smallest probability of infection. The probabilities of virus shedding from infected individuals by genotypes ranged from 50.0–83.3%, and it was shown that the virus shedding frequency of GI and GII were about the same.

**Table 1.** Estimated risk of infection (*p*), asymptomatic infection given successful infection (*s*) and virus positive outcomes of stool sample (*q*)

Grouped genotypes	Risk of infection (%)	Risk of asymptomatic infection (%)	Risk of virus positive outcome (%)
GI+GII (All)	25.4 (23.4, 27.4)	32.1 (27.7, 36.7)	73.2 (68.6, 77.4)
GI (All)	45.2 (32.6, 61.8)	44.0 (25.8, 63.3)	70.0 (48.3, 86.8)
GI 3	58.8 (29.4, 141.6)	50.0 (15.6, 84.4)	60.0 (19.9, 91.9)
GI 14	75.0 (24.3, NA)	66.7 (16.1, 97.7)	50.0 (3.8, 96.2)
GII (All)	24.7 (22.7, 26.7)	31.4 (26.9, 36.1)	73.4 (68.7, 77.7)
GII 1	60.0 (34.2, 94.6)	37.5 (11.0, 71.0)	83.3 (44.6, 99.0)
GII 2	93.8 (58.6, 146.8)	33.3 (6.5, 71.9)	80.0 (37.2, 98.7)
GII 3	34.9 (27.2, 43.6)	22.2 (10.8, 37.4)	62.2 (47.7, 75.4)
GII 4	18.4 (15.9, 21.3)	40.7 (32.8, 49.0)	68.0 (59.4, 75.9)

Profile likelihood based 95% confidence intervals are shown in parentheses.

NA (in an upper uncertainty bound) stands for the result which could not converge in our computing due to the sample size.

**Figure 3.**
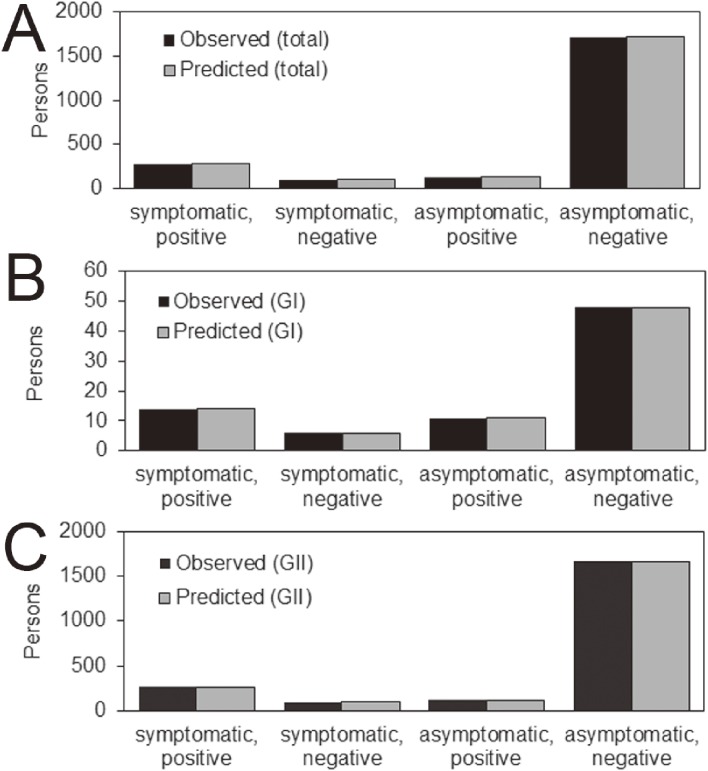
Comparison between observed and predicted number of people by symptom and PCR testing result. A) Results from genogroup-combined model with three parameters. B and C) Genogroup-separated modelling results with a total of six parameters.

Calculating the AIC for each interpretation scenario, the AIC of the genogroup-combined model (ie, a model with three parameters only) yielded the value of 1,227.9, while that of the genogroup-separated model (ie, a model with six parameters) was 1,177.4. The smaller value of AIC for a genogroup-separated model indicated that the model with genogroup information was better fitted to outbreak data, so there might potentially be a difference between GI and GII. Figure 3 compared observed and predicted numbers of people by symptom and PCR testing result, visually demonstrating the satisfactory description of observed patterns. A χ^2^ goodness-of-fit test revealed no significant deviations between predicted and observed numbers for both genogroup-combined and genogroup-separated models (*P* > 0.500 for both models with degrees of freedom at 3 and 7, respectively).

**Figure 4.**
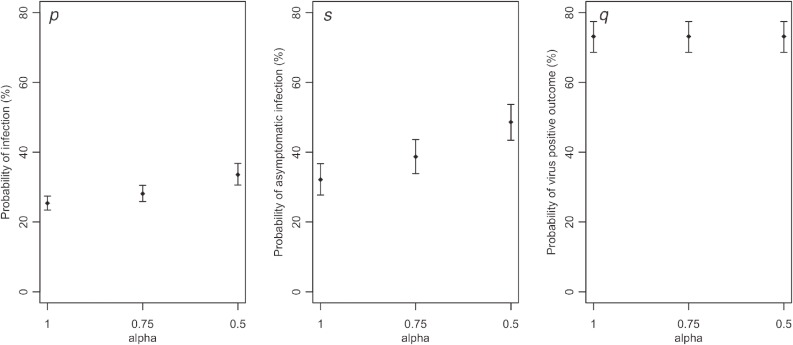
Sensitivity of estimated probabilities to the relative risk (alpha) of virus shedding among asymptomatic infected individuals compared with symptomatic cases. The value of alpha, defined as the ratio of the probability of shedding virus by an asymptomatic individual to the one by a symptomatic individual, was varied from 0.5 to 1.0 (baseline).

Varying the value of α from 0.50 to 1.00, we examined the sensitivity of model parameters to differential virus shedding frequency between symptomatic and asymptomatic infections (Figure 4). When α gets smaller, both the risk of infection (*p*) and the asymptomatic ratio (*s*) were estimated to be greater. The probability of virus shedding among symptomatic cases remained stable (Figure 4C).

Of the total of 55 outbreaks, there were two outbreaks involving large number of subjects (*n* = 1,328 for two outbreaks; 59.6% of the total), which could lead us to feel that the overall results may be highly influenced by those two outbreaks. Removing the two outbreaks and analyzing the remaining 53 altogether, parameters *p*, *s*, and *q* were estimated at 44.0% (95% CI, 40.5–47.7%), 25.9% (95% CI, 21.0–31.1%) and 73.1% (95% CI, 67.9–78.0%), respectively. The risk of infection (*p*) was significantly greater than the estimate in which all 55 outbreaks were analyzed together, but two other parameters, including asymptomatic ratio (*s*) and the risk of virus shedding (*q*), were not significantly deviated.

## DISCUSSION

The present study reanalyzed foodborne norovirus outbreaks in Japan from 2005–2006 that involved laboratory testing of all individuals including healthy people. To statistically infer asymptomatic ratio in joint with the risk of infection and the probability of virus shedding, a statistical model was developed to describe the data generating process. Assuming that all 55 outbreaks occurred due to repeated random samplings, the risk of infection (*p*) and the asymptomatic ratio (*s*) were estimated to be 25.4% and 32.1%, respectively. The probability of virus shedding (*q*) was estimated at 73.2%. Possible differences by genogroup were implicated by penalized likelihoods. The risk of infection was small for GII.4, while the asymptomatic ratio was estimated to be high for this common genotype.

An important contribution of the present study to the literature of norovirus is that the asymptomatic ratio was estimated to be about 30%, which is consistent with existing literature based on volunteer challenge studies.^5^^,^^8^^,^^26^ Not based on human volunteer challenge, our study has successfully estimated the asymptomatic ratio from naturally occurred outbreaks, as has also been achieved for other infectious diseases.^34^ In particular, the asymptomatic ratio of common genotype GII.4 was estimated to be high, which is in line with the conventional understanding that the control of GII.4 is likely more difficult than that of other genotypes. In addition to original findings of similar virus shedding frequency of GII.4 between symptomatic and asymptomatic cases by Ozawa et al,^31^ our study underscores the important feature of GII.4 inducing asymptomatic infections among food handlers. Not only spreading the virus via food handlers, but the greater asymptomatic ratio allows infected individuals to be freely mobile and have more opportunities to pass the virus to others. When we assumed that the actual frequency of virus shedding from asymptomatic individuals is smaller than symptomatic cases, the asymptomatic ratio was estimated to be greater than the baseline. In other words, the estimated value around 30% might be even greater than our result.

There was no statistically significant difference between GI and GII, while the AIC value was indicative that there might be a difference between GI and GII. This was caused by limited sample size, and slight non-significant differences (eg, greater risk of infection for GI than GII and smaller asymptomatic ratio for GI than GII) were identified, implying that GI might be more controllable than GII. To our knowledge, the present study is the first to epidemiologically endorse different patterns of outbreaks by genogroups in natural settings, though differences have been indicated in experimental studies.^12^^,^^22^^,^^23^ While a greater asymptomatic ratio for GII.4 than others was consistent with our existing understanding, the smaller infection risk with GII.4 than others calls for careful interpretation. Due to the frequent circulation of GII.4, a portion of exposed individuals might have been immune in advance of outbreaks (that were not widespread before 2006 but may have remained unrecognized); moreover, evolutionary changes in GII.4 (eg, antigenic evolution) might have later varied the virus characteristics, including the infectiousness of GII.4. Evolutionary epidemiological study of GII.4 to be adapted to human populations is a subject of our ongoing study.

Two limitations must be discussed. First, the survey data contained epidemiologically limited information. That is, to define symptomatic cases, diarrhea or vomiting was employed, but they were qualitative definitions that did not explicitly involve the frequency, such as the number of defecations/vomiting per day. Such qualitative definition is favored for clearly defining what a symptomatic case is, and an important advantage of the present study is that an identical research group has conducted the entire survey and minimized the measurement of error that could potentially arise from researchers. Nevertheless, when it comes to additionally measuring the severity of illness, which is out of the scope of the present study, it might have been unavoidable to include human perception error. Such misclassification could have potentially affected the asymptomatic ratio (eg, due to broad definition of asymptomatic persons). Moreover, the exact timing of stool sampling was unavailable; however, it is likely that all sampling took place in a matter of 1 week from the onset of outbreaks, during which the virus shedding level of both symptomatic and asymptomatic cases should be substantial.^8^ Second, the model involved a few simplifications. The simplified points include the ignorance of the dependent happening in the risk of infection. Since the majority of outbreaks were considered as purely foodborne, a single parameter *p* was allocated, but if human-to-human transmission was involved, it might have been better to account for that information, which was not attainable in the present study using the same dataset. Another simplification was the use of binomial sampling process for each likelihood. As a consequence, the uncertainty bound of *p* in the present study should be regarded as an underestimate. One of the drawbacks has been seen in significantly greater *p* that we obtained when two big outbreaks were removed from analysis; in other words, the estimate might not have been significantly different if the uncertainty also reflected demographic stochasticity.

Despite the abovementioned limitations, our study has successfully demonstrated that the asymptomatic ratio can be estimated from outbreak data in which all involved individuals undertook laboratory testing and also that the asymptomatic ratio was about 30% in natural outbreak settings. Practical difficulty in controlling GII.4 outbreaks was supported by its large estimate of the asymptomatic ratio. Collecting similar datasets from further outbreaks, we will be able to gain further insights into the natural history of norovirus infection, including genotype-specific differences. Understanding the natural history better, similar explicit estimates based on natural infection in future could help clarify better control strategies of norovirus outbreaks, even by genotype.

## APPENDIX A. SUPPLEMENTARY DATA

eFigure 1. Estimated probabilities of infection, asymptomatic infection given successful infection and virus positive outcomes of stool sample. Dots show the maximum likelihood estimates, while whiskers extend from lower to upper 95% confidence intervals derived from the profile likelihood. Horizontal axis groups specific genotypes in common.

eTable 1. Counts of symptomatic and asymptomatic cases with virological testing results for each outbreak.
