# Supplementary material for: Estimating the Asymptomatic Ratio of Norovirus Infection During Foodborne Outbreaks With Laboratory Testing in Japan
Source: J Epidemiol. 2018 Sep 5;28(9):382–7. doi: 10.2188/jea.JE20170040 (PMC6111106; doi:10.2188/jea.JE20170040)
Supplement: Supplementary file 1 [file je-28-382-s001.pdf]

eTable 1. Counts of symptomatic and asymptomatic cases with virological testing results for each outbreak.

sym_pos	sym_all	asym_pos	asym_all
2	3	0	30
3	3	1	22
37	42	24	425
6	7	0	14
2	2	0	5
2	2	0	0
7	8	3	21
1	2	1	2
2	2	0	0
4	6	3	5
2	3	1	3
4	8	2	9
2	2	0	6
0	2	2	12
0	1	2	7
2	2	1	10
0	0	2	5
11	12	0	0
2	8	0	0
4	6	4	53
29	48	34	813
2	2	2	19
1	1	1	15
2	4	4	32
6	6	2	35
6	11	7	31
1	1	2	14
3	3	2	8
2	2	0	0
2	4	1	18
3	3	0	0
1	2	1	4
3	4	2	11
33	41	0	0
0	0	2	8
3	3	0	14
3	5	1	11
9	18	0	0
30	35	2	3
2	3	0	0
1	1	1	9
8	11	0	0

2	4	0	2
1	3	1	6
1	3	2	6
1	2	1	7
4	5	2	3
7	10	2	4
4	4	2	3
4	4	2	81
2	2	3	6
3	5	0	0
1	2	2	6
3	5	3	12
5	6	3	35

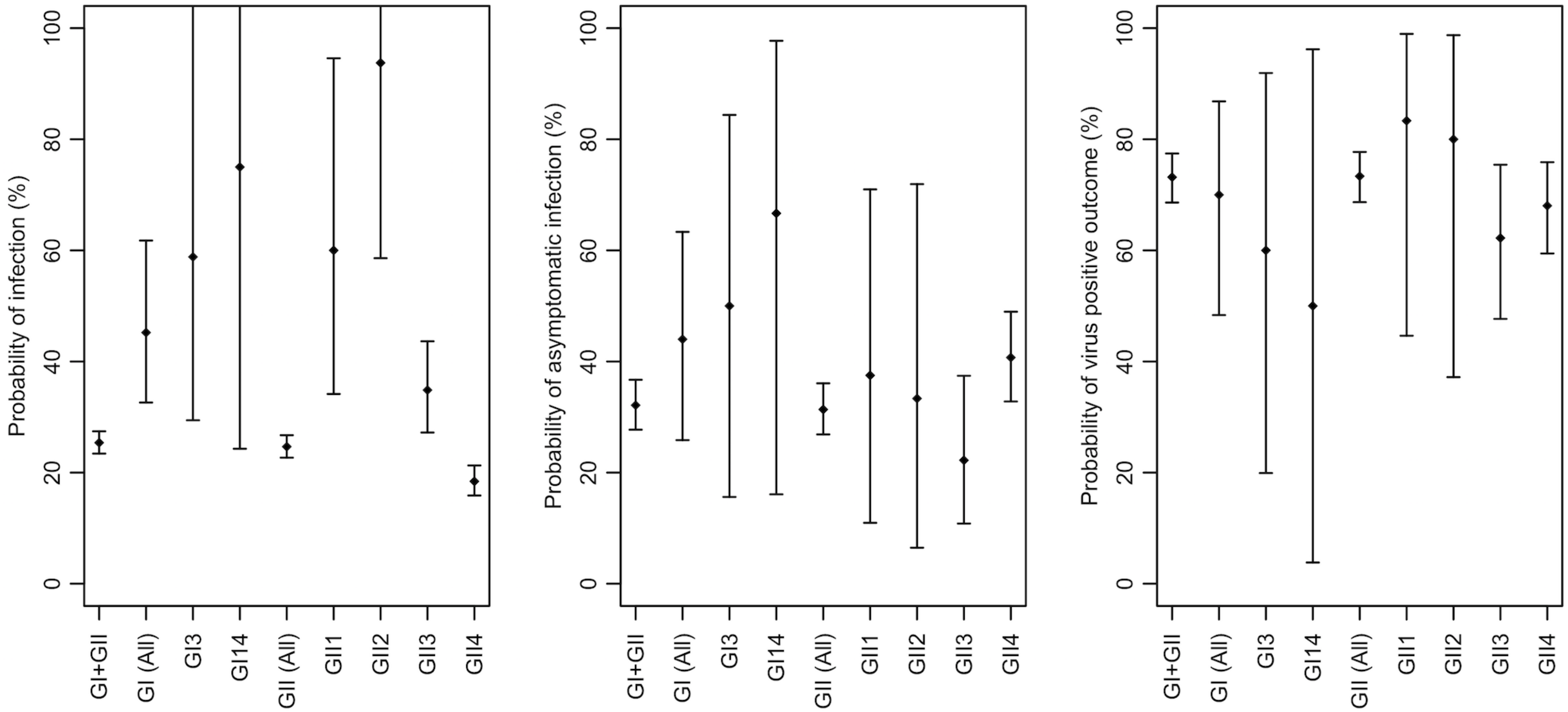

eFigure 1. Estimated probabilities of infection, asymptomatic infection given successful infection and virus positive outcomes of stool sample. Dots show the maximum likelihood estimates, while whiskers extend from lower to upper 95% confidence intervals derived from the profile likelihood. Horizontal axis groups specific genotypes in common.
